# Supplementary material for: Alkaline Soil Degradation and Crop Safety of 5-Substituted Chlorsulfuron Derivatives
Source: Molecules. 2022 May 21;27(10):3318. doi: 10.3390/molecules27103318 (PMC9145588; doi:10.3390/molecules27103318)
Supplement: Supplementary file 1 [file molecules-27-03318-s001.zip › molecules-1686247-supplementary.pdf]

Table S1. The Herbicidal Activity of Target Compounds L101-L107.

Compound	Concentration (g·ha $^{-1}$)	Herbicidal activity (%)							
		Brassica campestris		*Amaranthus tricolor*		*Echinochloa crusgalli*		*Digitaria sanguinalis*	
		Pre	*Post*	*Pre*	*Post*	*Pre*	*Post*	*Pre*	*Post*
**Chlorsulfuron**	30	100	100	99.2	100	67.9	79.2	14.6	0
	150	100	100	100	100	88.0	84.7	69.9	0
**L101**	30	65.7	90.7	56.9	80.0	53.4	100	0	10.5
	150	73.4	100	97.2	87.2	84.1	100	11.4	30.6
**L102**	30	17.6	44.9	84.8	100	40.7	35.8	0	9.6
	150	32.7	62.1	88.6	100	50.6	47.5	10.7	13.9
**L103**	30	100	100	100	100	57.2	59.7	1.0	3.8
	150	100	100	100	100	72.8	64.4	8.7	8.1
**L104**	30	64.2	100	82.6	99.2	20.2	15.4	0	9.6
	150	87.7	100	97.0	100	33.3	33.8	36.9	39.7
**L105**	30	54.1	58.6	52.3	100	8.6	11.1	14.6	0
	150	95.6	75.8	80.3	100	29.2	18.1	30.1	16.7
**L106**	30	30.1	42.2	0	38.9	12.5	14.8	0	0
	150	44.1	65.8	13.9	59.6	13.6	62.7	0	6.1
**L107**	30	95.6	100	100	100	19.3	36.9	29.1	0
	150	97.5	100	100	100	59.7	55.7	39.8	16.7

SI1-Soil degradation assay

Soil degradation research was conducted by the following steps.

Soil selection: The soil was derived from the upper-layer (0–25 cm) in fresh farm land, and air-dried in the shade, shifted through 2 mm sieve according to the Chinese National Standard GB/T 31270.1-2014.[1] The soil texture, pH value, organic matter, cation exchange capacity (CEC) and particle size analysis of tested alkaline soil were determined by Tianjin Institute of Agricultural Resources and Environment Science.

HPLC conditions: The target compounds were explored and standard curves were established according to the Chinese National Standard GB/T 16631-2008.[2] Chromatographically pure methanol, acetonitrile and ultrapure water (pH 3.0) were used as the mobile phase. The retention time should remain between 10-20 minutes.

Standard curve: The standard curves used for quantitative conversion were established with the injection volume of 10 μL at 20 °C. The concentration range of the standard curve was between 200 $ug{\cdot}mL^{-1}$ and 0.025 $ug{\cdot}mL^{-1}$ at 20 °C.

Measurement of the Recovery Rate: According to the Chinese National Standard GB/T 31270.1-2014, the Chinese Agricultural Industry Standard NY/T788-2004 and the Chinese Agricultural Industry Standard NY/T788-2018,[3-4] the concentration of the test compounds in 20 g of soil in a 100 mL conical flask were 5 $mg{\cdot}kg^{-1}$, 2 $mg{\cdot}kg^{-1}g$, and 0.5 $mg{\cdot}kg^{-1}$ (adjusted with an acetonitrile solution), respectively. Each concentration was repeated 5 times and recovery rate should range from 70% to 110% and guarantee the coefficient of variation <5% to ensure the reliability of the method.

The extraction method: The regulation of 60% water holding capacity (4 mL) after the acetonitrile evaporated completely (about 5 min) was implemented and the soil samples were mixed well. Suitable extraction solvent was added into the flask and then was shook in the thermostatic oscillator for 3 h at 200 rpm/min. The samples were centrifuged at 6500 rpm in a Thermo Scientific centrifuge at 20℃ for 2 min. The supernatant liquid were combined and concentrated. Then dichloromethane (30 mL×2) and 30 mL of HPLC grade water were used for the extraction of the residues. The organic phase were combined and dried by anhydrous sodium sulfate. And then the mixture were filtered and concentrated at 25℃. The concentrated samples were dissolved into 10 mL acetonitrile and shook in oscillator at room temperature for 1 h. The solutions were filtered through millipore filter (organic, nylon-66, 0.22 μm) for HPLC analysis.

Cultivation of samples and management: Each sample with its concentration at 5 $mg{\cdot}kg^{-1}$ was added, 60% water holding capacity was then regulated. The sealed soil samples were cultivated in a biochemical incubator at 25±1℃ and 80% humidity in the dark. The degradation curves followed the first order kinetic equation $Ct = C_0 \times e^{-kt}$. DT_{50} were calculated according to the formula: $DT_{50}=\ln2/k$ and the statistical analysis was also guaranteed by the triplicated data.

References

1. The Institute for the Control of Agrochemicals under the Ministry of Agriculture. *Chinese National Standard GB/T 31270.1-2014. Test Guidelines on Environmental Safety Assessment for Chemical Pesticides—Part 1: Transformation in Soils;* Oct 10, **2014**.
2. National Chemical Standardization Technical Committee. *Chinese National Standard GB/T 16631-2008. General rules for high performance liquid chromatography*; Jun 18, **2008**.
3. Ministry of Agriculture, Pesticide Testing Center. *Chinese Agricultural Industry Standard NY/T 788-2004. Guideline on pesticide residue trials*; Apr 16, **2004**.

4. Ministry of Agriculture, Pesticide Testing Center. *Chinese Agricultural Industry Standard NY/T 788-2018. Guideline on pesticide residue trials*; July 27, **2018**.

SI2-Crop safety

Wheat safety test[1-3]

The wheat was dealt with selected compound through pre- and post-emergence treatments via pot trials in greenhouse (25 ± 2 °C). The applied concentration was 30 and 60 $g{\cdot}ha^{-1}$, and each treatment was triplicated. For pre-emergence treatment, the compound was sprayed onto the soil in cups (d=7 cm) following the seeding performance. After that, the moisture was maintained and cultivated in greenhouse. The fresh weight of the cover crop was measured after 22 days, and the safety was represented by the inhibition rate. The variance analysis of the data was performed by the Duncan multiple comparisons with the SPSS 22.0 software.

For post-emergence treatment, the seeding was conducted following adjusting the moisture and temperature until the wheat grew to 4-leaf stage. The compound was sprayed, and the result was measured after 28 days, the same procedure as pre emergence treatment.

Corn safety test

The corn safety was conducted through pre- and post-emergence treatments as wheat. After pre-emergence treatment of the relevant concentration of selected compound in cups (d=12 cm), the seeding was performed following the moisture and temperature adjustment. The result was determined after 16 days, and variance analysis was proceeded as wheat safety.

For post emergence treatment, the seeding was conducted until the corn grew to 3-leaf stage. The compound was sprayed, and the result was measured after 23 days, the same procedure as pre-emergence treatment.

The data analysis of variance was completed by Duncan multiple comparison using SPSS 22.0, a statistical analysis software for analysis of variance, regression analysis and correlation analysis etc.

References

1. Zhou, S.; Meng, F. F.; Hua, X. W.; Li, Y. H.; Liu, B.; Wang, B. L.; Chen, J.; Chen, A. L.; Li, Z. M., Research on Controllable Degradation of Novel Sulfonylurea Herbicides in Acidic and Alkaline Soils. J. Agric. Food Chem. 2020, 68 (10), 3017-3025.
2. Meng, F. F.; Wu, L.; Gu, Y. C.; Zhou, S.; Li, Y. H.; Chen, M. G.; Zhou, S.; Zhao, Y. Y.; Ma, Y.; Li, Z. M. Research on the Controllable Degradation of N-methylamido and Dialkylamino Substituted at the 5th Position of the Benzene Ring in Chlorsulfuron in Acidic Soil. RSC Adv. 2020, 10 (30), 17870-17880.
3. Wu, L.; Gu, Y.-C.; Li, Y.-H.; Meng, F.-F.; Zhou, S.; Li, Z.-M. Degradation of 5-DialkylaminoSubstituted Chlorsulfuron Derivatives in Alkaline Soil. Molecules 2022, 27, 1486. https:// doi.org/10.3390/molecules27051486.

SI3-Report of soil analysis in English

Tianjin Institude of Agriculture Resource and Environment

Analysis Report

Rwquester	Nankai University	Report number	HJ-F-FX-202012-013
Sample name	Soil samples	Date of receipt	December 3th, 2020
		Date of report	January 6th, 2021
Analysis items	Mechancal composition,pH,Organic matter,Cation exchange capacity		
Number of samples		Soil 1	Soil 2
pH		8.39	5.46
Cation exchange capacity (cmol⁺/kg)		7.3	14.4
Organic matter(g/kg)		19.4	8.37
Sample status		Brown ,lupm	Brown ,lupm
Mechancal composition	Texture class(g/kg)		
Soil 1	1-2mm(g/kg)		7.95
	0.5-1mm(g/kg)		24.6
	0.25-0.5mm(g/kg)		23.3
	0.05-0.02mm(g/kg)		79.0
	0.02-0.002mm(g/kg)		286
	＜0.02mm(g/kg)		282
	0.25-0.05mm(g/kg)		297
	2.0-0.05mm(g/kg)		353
	0.05-0.002mm(g/kg)		365
Soil 2	1-2mm(g/kg)		0.750
	0.5-1mm(g/kg)		3.81
	0.25-0.5mm(g/kg)		7.08
	0.05-0.02mm(g/kg)		125
	0.02-0.002mm(g/kg)		179
	＜0.02mm(g/kg)		105
	0.25-0.05mm(g/kg)		579
	2.0-0.05mm(g/kg)		591
	0.05-0.002mm(g/kg)		304

SI4-Report of soil analysis in Chinese

170012051117

监 测 报 告

报告编号：HJ-F-FX-202012-031

委 托 单 位　　南开大学

委托单位地址　　天津市南开区卫津路94号

监 测 内 容　　土壤分析

天津市生态环境监测中心（盖章）

报告说明

1、报告无本中心报告专用章、骑缝章无效。

2、对于非本中心人员采集的样品，结果仅对客户送检样品结果负责。

3、对现场不可复现的样品，仅对采样所代表的时间和空间负责。

4、未经书面授权，不得部分复制本报告。

5、未经本中心同意，该监测报告不得用于商业性宣传。

6、因客户提供的信息有误，影响监测结果的有效性时，造成的一切后果与本中心无关。

地　　址：天津市南开区复康路19号

电　　话：022-87671699

传　　真：022-87671699

邮政编码：300191

电子邮箱：temcjcglb@tj.gov.cn

送检日期：2020年12月3日

分析日期：2020年12月07日-2020年12月08日

方法和仪器：

项目	方法及依据	仪器名称、型号和编号
pH值	土壤 pH值的测定 电位法（HJ 962-2018）	SevenExcellence S500-k pH（酸度）计(B546705501)<br>CPA225D型 电子分析天平(24190372)
有机质	土壤检测 第6部分：土壤有机质的测定 NY/T1121.6-2006	滴定管(滴定管-分-02)<br>BSA124S型 电子分析天平(29490274)
阳离子交换量	土壤 阳离子交换量的测定 三氯化六氨合钴浸提 分光光度法（HJ 889-2017）	DR6000 双光束紫外可见分光光度计(1492931)<br>OHAOS AR2140 电子天平(1201310779)
机械组成（质地）	森林土壤颗粒组成（机械组成）的测定密度计法（LY/T 1225-1999）	TM-85土壤密度计(341)<br>BSA124S型 电子分析天平(29490274)

项目及结果：

结果 项目(单位) / 样品名称	pH值（无量纲）	阳离子交换量（$cmol^+$/kg）	有机质（g/kg）	机械组成（质地）	样品状态描述
土1	8.39	7.3	19.4	粘壤土	褐色块状
土2	5.46	14.4	8.37	砂质壤土	褐色块状

备注：
1. 监测方法为客户指定。
2. 样品名称为客户提供信息，中心对此真实性不承担责任。

编制人：[illegible]　　　　审核人：[illegible]

签发人：[illegible]　　　　签发日期：2021年1月6日

附表

样品名称	土壤机械组成（各粒级含量）	
土1	1-2mm 粒级含量（g/kg）	7.95
	0.5-1mm 粒级含量（g/kg）	24.6
	0.25-0.5mm 粒级含量（g/kg）	23.3
	粉（砂）粒　0.05-0.02mm 粒级含量（g/kg）	79.0
	粉（砂）粒　0.02-0.002mm粒级含量（g/kg）	286
	粘粒<0.002mm粒级含量（g/kg）	282
	细砂+极细砂0.25-0.05mm粒级含量（g/kg）	297
	砂粒2.0-0.05mm粒级含量（g/kg）	353
	粉(砂)粒0.05-0.002mm粒级含量（g/kg）	365
土2	1-2mm 粒级含量（g/kg）	0.750
	0.5-1mm 粒级含量（g/kg）	3.81
	0.25-0.5mm 粒级含量（g/kg）	7.08
	粉（砂）粒　0.05-0.02mm 粒级含量（g/kg）	125
	粉（砂）粒　0.02-0.002mm粒级含量（g/kg）	179
	粘粒<0.002mm粒级含量（g/kg）	105
	细砂+极细砂0.25-0.05mm粒级含量（g/kg）	579
	砂粒2.0-0.05mm粒级含量（g/kg）	591
	粉(砂)粒0.05-0.002mm粒级含量（g/kg）	304
